# AMPK Phosphorylation Is Controlled by Glucose Transport Rate in a PKA-Independent Manner

**DOI:** 10.3390/ijms22179483

**Published:** 2021-08-31

**Authors:** Riccardo Milanesi, Farida Tripodi, Jacopo Vertemara, Renata Tisi, Paola Coccetti

**Affiliations:** Department of Biotechnology and Biosciences, University of Milano-Bicocca, 20126 Milano, Italy; r.milanesi2@campus.unimib.it (R.M.); jacopo.vertemara@unimib.it (J.V.); renata.tisi@unimib.it (R.T.)

**Keywords:** Snf1/AMPK, *Saccharomyces cerevisiae*, glucose-6-phosphate, hexose transport, protein–metabolite interaction

## Abstract

To achieve growth, microbial organisms must cope with stresses and adapt to the environment, exploiting the available nutrients with the highest efficiency. In *Saccharomyces cerevisiae*, Ras/PKA and Snf1/AMPK pathways regulate cellular metabolism according to the supply of glucose, alternatively supporting fermentation or mitochondrial respiration. Many reports have highlighted crosstalk between these two pathways, even without providing a comprehensive mechanism of regulation. Here, we show that glucose-dependent inactivation of Snf1/AMPK is independent from the Ras/PKA pathway. Decoupling glucose uptake rate from glucose concentration, we highlight a strong coordination between glycolytic metabolism and Snf1/AMPK, with an inverse correlation between Snf1/AMPK phosphorylation state and glucose uptake rate, regardless of glucose concentration in the medium. Despite fructose-1,6-bisphosphate (F1,6BP) being proposed as a glycolytic flux sensor, we demonstrate that glucose-6-phosphate (G6P), and not F1,6BP, is involved in the control of Snf1/AMPK phosphorylation state. Altogether, this study supports a model by which Snf1/AMPK senses glucose flux independently from PKA activity, and thanks to conversion of glucose into G6P.

## 1. Introduction

Every living organism must adapt to challenging environments, coping with stresses and monitoring the availability of nutrients. For microbial cells, the success of this adaptation is represented by the ability to grow, such that the maximization of the growth rate is usually taken as the objective function of a cell [1]. To this aim, the fine-tuning of several cellular processes is pivotal for the coordination of metabolism, cell cycle, and growth. In this process, the crosstalk between different signaling pathways is necessary to successfully coordinate cell functions, as highlighted by the widespread bow-tie architecture of signal transduction pathways [2]. 

The budding yeast *Saccharomyces cerevisiae* is a powerful model for studying fundamental aspects of eukaryotic cell biology, especially connected to signal transduction and metabolism, thanks to the high degree of conservation of several key cellular processes between yeast and mammalian cells [3]. Its metabolism is characterized by the preferential aerobic fermentation of glucose, similarly to Warburg-positive transformed cells. In yeast cells, glucose availability is perceived by Snf3/Rgt2 and Gpr1/Gpa2 systems, activates the Ras/PKA pathway, and suppresses the Snf1/AMPK pathway [4]. This results in the promotion of glucose fermentation and cell growth, while stress tolerance, mitochondrial respiration, and the consumption of alternative carbon sources to glucose are repressed [5]. On the other hand, low glucose concentration is associated with the repression of the Ras/PKA pathway and the activation of Snf1/AMPK, leading to the induction of genes associated with respiration, β-oxidation, and the upregulation of stress tolerance [6].

Among the genes controlled by glucose availability, there are those required for glucose transport. *S. cerevisiae* possesses at least 20 different glucose transporters, all presenting a common structure with 12 transmembrane regions, and characterized by different affinity and capacity parameters [4]. Yeast specialization for glucose consumption is highlighted by its ability to dynamically adapt the expression pattern of glucose transporters according to the extracellular glucose concentration. High titration leads to the expression of low-affinity and high-capacity transporters (such as Hxt1 and Hxt3), allowing for a massive intake flux. Instead, a decrease in glucose concentration leads to the expression of high-affinity and low-capacity transporters (such as Hxt7, Htx6 and Gal2), succeeding in the passive import of glucose even at low millimolar concentrations [4]. 

The PKA and Snf1/AMPK pathways’ opposite activations suggest the existence of a thick crosstalk between these two pathways. Indeed, Snf1/AMPK phosphorylates and inhibits Cyr1—the adenylate cyclase upstream of the PKA pathway [7]. On the other hand, PKA is known to phosphorylate and inactivate Sak1—the major activator kinase of Snf1/AMPK [8]. This feedback mechanism—by which PKA inactivates Snf1/AMPK, and vice versa, according to nutritional conditions—allows yeast cells to avoid transcriptional inconsistencies undermining cellular homeostasis. In any case, the molecular mechanism underlying this crosstalk is still missing [9].

Yeast cells selectively consume different carbon sources through respiration or fermentation. It has been shown that sugar uptake rate defines the manner of their consumption, going beyond the classical model by which yeast’s metabolic fate is controlled by glucose concentration in the growth medium [10]. Moreover, fructose-1,6-bisphosphate (F1,6BP) concentration has been reported to be correlated with sugar transport rate, mirroring the glycolytic flux and potentially acting as a flux sensor [10,11]. Following this line of evidence, F1,6BP could couple the glycolytic rate with the switch between respiration and fermentation, giving rise to a flux-sensing mechanism. Several reports also suggest that the coordination of cell metabolism and signaling takes place through protein–metabolite interactions (PMIs) between key molecules of the central metabolism and proteins belonging to different signaling pathways [12]. Interestingly, F1,6BP has been reported to interact with Cdc25—the guanine nucleotide exchange factor upstream of Ras—inducing the activation of the Ras/PKA pathway [13]. This interaction connects PKA with the function of F1,6BP as a reporter of the glycolytic flux, suggesting a molecular regulatory role of the flux-sensing mechanism. Strikingly, the Ras/PKA pathway is also responsible for the activation of Reg1/Glc7—the phosphatase complex upstream of Snf1/AMPK—thus leaving open the hypothesis that Snf1/AMPK activation state may be indirectly linked to glucose metabolism through F1,6BP synthesis and PKA activation.

In keeping with this hypothesis, mammalian AMPK has recently been proven to be controlled by F1,6BP, which induces the release of AMPK from the AXIN/LKB1/AMPK complex [14]. In this process, signal transduction from glycolysis to AMPK is mediated by F1,6BP’s interaction with aldolase, whose activity is essential for F1,6BP-dependent control of the AMPK activation state [14]. However, the *S. cerevisiae* ortholog of the AXIN/LKB1/AMPK complex has not been identified, suggesting a different coordination between glycolysis and Snf1/AMPK activity.

In the present paper, we investigate whether the connection between Snf1/AMPK and glycolytic metabolism is indirectly mediated by the Ras/PKA pathway. Interestingly, we demonstrate that glucose-6-phosphate (G6P), and not F1,6BP, is involved in the control of the Snf1/AMPK phosphorylation state. We also report that the Ras/PKA pathway is dispensable for the inhibition of Snf1/AMPK upon glucose replenishment. Therefore, we propose that the glycolytic flux is sensed by different signaling pathways (i.e., Snf1/AMPK and Ras/PKA) independently, and through interactions with different glycolytic metabolites (i.e., G6P and F1,6BP), to achieve the proper cellular response to the specific nutrient condition.

## 2. Results

### 2.1. Glucose Inhibition of Snf1/AMPK Is Independent from the Ras/PKA Pathway

The Snf1/AMPK activation state is controlled by phosphorylation of the regulatory residue Thr210—a substrate of both the upstream kinases (Sak1, Tos3, and Elm1) and the Reg1/Glc7 phosphatase [15,16]. Glucose availability promotes Snf1-T210 dephosphorylation, while growth on non-fermentable carbon sources is associated with a high level of Snf1/AMPK phosphorylation. As already reported, PKA phosphorylates Sak1, inducing a mild reduction in Snf1/AMPK phosphorylation [8]. According to another report, the activity of Snf1/AMPK upstream kinases is not influenced by glucose availability [17]. In addition, Reg1/Glc7 phosphatase is activated by glucose in a Ras/PKA-dependent manner [16]. Taken together, all of these observations prompted us to better investigate the involvement of the Ras/PKA pathway in the control of the phosphorylation state of Snf1/AMPK.

**Figure 1:**
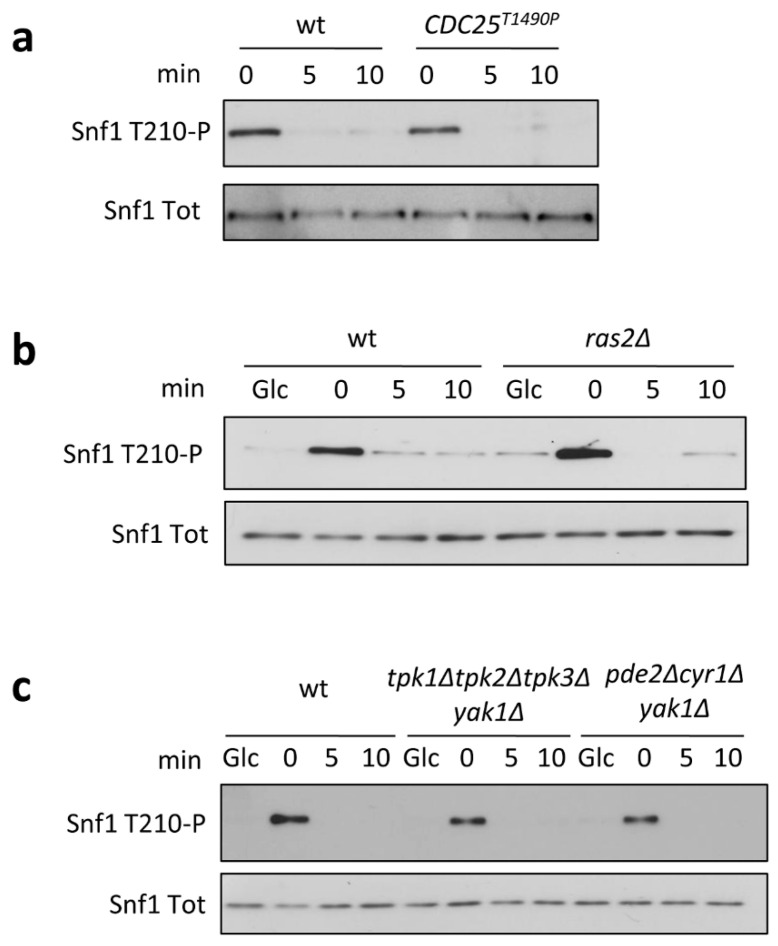
Snf1-T210 phosphorylation is independent from the Ras/PKA pathway. (**a**–**c**) Shift-up experiments in (**a**) wild-type and *CDC25^T1490P^* strains, (**b**) *ras2Δ* strain, and (**c**) strains lacking PKA activity (*tpk1∆tpk2∆tpk3∆yak1∆* and *pde1∆cyr1∆yak1∆*). One representative Western blot analysis using anti-pAMPK antibody (to detect Snf1-T210 phosphorylation) and anti-His antibody (to detect total Snf1) is shown.

Since the Ras/PKA pathway is linked to glycolysis by F1,6BP physical interaction with Cdc25 [13], we first asked whether Snf1/AMPK phosphorylation may be indirectly influenced by this interaction. To address this issue, we carried out shift-up experiments, growing yeast cells in ethanol (a condition in which Snf1/AMPK phosphorylation is high) and then adding glucose at the final concentration of 2% (which induces a rapid Snf1/AMPK dephosphorylation). Snf1/AMPK phosphorylation rapidly decreased in the wild-type strain, becoming barely detectable 5 min after the addition of glucose (Figure 1a). The same behavior was observed in the *CDC25^T1490P^* mutant, known to prevent F1,6BP-dependent activation of Ras [13] (Figure 1a). To further dissect the involvement of the Ras/PKA pathway, we decided to test the effect of the lack of the Ras2 protein on Snf1/AMPK dephosphorylation, carrying out a shift-up experiment in a *ras2∆* strain. As in the *CDC25^T1490P^* strain (Figure 1a), we also observed complete dephosphorylation of Snf1/AMPK after 5 min in the *ras2∆* strain (Figure 1b), thus excluding the possibility that Snf1/AMPK could indirectly perceive the availability of glucose through F1,6BP stimulation of the Ras/PKA pathway.

As reported previously, Ras/PKA activity is pivotal for glucose induction of PP1 and PP2A phosphatases [16]. The glucose-dependent Snf1/AMPK dephosphorylation in the *ras2∆* strain shown in Figure 1b can either depend on residual Ras activity due to the presence of Ras1, still able to activate the PKA pathway [18], or be completely independent from the PKA-dependent phosphatase activation. To discern between these two possibilities, we carried out shift-up experiments in the *tpk1∆tpk2∆tpk3∆yak1∆* strain, which completely lacks PKA activity. We also confirmed that no defect in Snf1/AMPK dephosphorylation was observed in this background (Figure 1c), thus excluding any involvement of PKA activity in Snf1/AMPK inactivation upon glucose replenishment. 

Along with the glycolytic flux, the Ras/PKA pathway perceives extracellular glucose availability through the Gpr1/Gpa2 system, which integrates nutritional signals with the modulation of cell fate via cAMP synthesis and PKA activation [4]. Therefore, we cannot exclude the possibility that PKA activation triggered by extracellular glucose could influence the Snf1/AMPK phosphorylation state. To rule this out, we performed a shift-up experiment in a *pde1∆cyr1∆yak1∆* strain, in which *CYR1* deletion prevents both intracellular and extracellular stimulation of PKA activity. Remarkably, as in the control strain, Snf1/AMPK was completely dephosphorylated after the shift-up (Figure 1c), supporting a model by which its phosphorylation state is controlled by glucose independently from the Ras/PKA pathway, in terms of both intracellular and extracellular perception of glucose. 

### 2.2. Snf1/AMPK Phosphorylation Is Correlated with Glucose Transport Rate

Snf1/AMPK phosphorylation has always been correlated with glucose availability. A high glucose concentration is associated with low phosphorylation of Snf1/AMPK, while high phosphorylation of Snf1/AMPK is a feature of cells grown in low glucose concentrations (lower than 0.05%) [19]. Moreover, different glucose concentrations are associated with alternative metabolic states of yeast cells. Indeed, a high glucose concentration supports fermentation, while a low one induces a respiratory metabolism.

Recently, yeast metabolism has been correlated with sugar transport rate, which induces fermentation in a high glucose flux and mitochondrial respiration in a low one [10]. Considering this change of paradigm from glucose concentration to its flux rate, we also asked whether Snf1/AMPK phosphorylation state could be controlled by glucose transport rate rather than glucose concentration in the medium.

**Figure 2:**
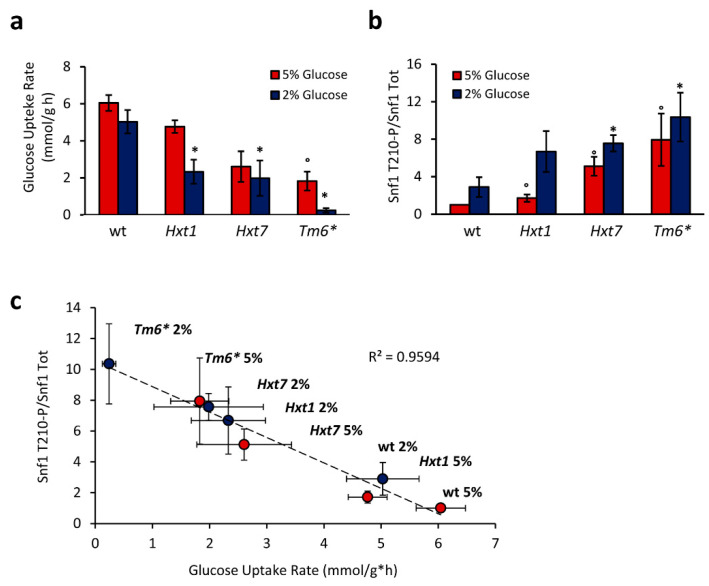
Snf1-T210 phosphorylation correlates with glucose transport rate. (**a**) Glucose uptake rate and (**b**) Snf1-T210 phosphorylation level measured in 2% and 5% glucose media of yeast strains expressing only Hxt1, Hxt7, or the chimeric transporter Tm6*, under a constitutive, non-glucose-responsive promoter. Mean ± standard deviation is shown (*n* = 3 for each). * *p* < 0.05 relative to wt in 2% glucose; ° *p* < 0.05 relative to wt in 5% glucose (*t*-test). (**c**) Linear correlation between Snf1 phosphorylation and glucose uptake rate shown in (**a**,**b**).

To address this issue, we used yeast strains expressing only one type of hexose transporter under a constitutive promoter: the high-capacity low-affinity Hxt1, the high-affinity low-capacity Hxt7, or the chimeric transporter Tm6*, composed of the N-terminal of Hxt1 and the C-terminal of Hxt7 [20]. As previously reported, these strains exhibited a reduction in glucose consumption rate, with the Tm6*-expressing strain showing the lowest transport rate in both 2% and 5% glucose-containing media [21] (Figure 2a). 

Interestingly, these strains also presented altered phosphorylation of Snf1/AMPK. In fact, all of the mutants showed an increased level of Snf1-T210 phosphorylation, in both 2% and 5% glucose media (conditions in which wild-type cells present a fermentative metabolism and a very low Snf1-T210 phosphorylation; Figure 2b). Altering the expression of glucose transporters, we managed to uncouple glucose transport rate from glucose concentration in the medium, observing complete independence of Snf1/AMPK phosphorylation from glucose concentration (Figure 2b). Indeed, we found an inverse correlation between Snf1/AMPK phosphorylation state and glucose consumption rate (Figure 2c), demonstrating that Snf1/AMPK activation state is not statically controlled by glucose availability, but rather dynamically regulated by transport flux. 

**Figure 3:**
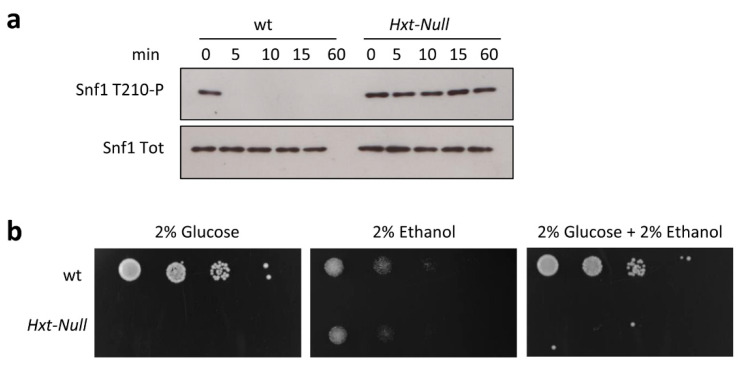
Snf1-T210 dephosphorylation requires glucose transport. (**a**,**b**) Shift-up experiments in wild-type and *Hxt-Null* strains. Cells were harvested after 0, 5, 10, 15, and 60 min of glucose supplementation; anti-pAMPK antibody (to detect Snf1-T210 phosphorylation) and anti-His antibody (to detect total Snf1/AMPK) were used; one representative immunoblot is shown. (**b**) Wild-type and *Hxt-Null* strains were equalized, spotted at 10-fold serial dilutions, and grown on synthetic complete plates containing 2% glucose, 2% ethanol, or 2% glucose and 2% ethanol as carbon sources.

To confirm that Snf1/AMPK activation depends on glucose transport, we carried out shift-up experiments using a strain deleted in all of the hexose transporters (*Hxt-Null*) and unable to grow on glucose due to its inability to import glucose from the medium [22]. The addition of glucose to ethanol-grown cells induced rapid and almost complete dephosphorylation of Snf1/AMPK in the wild-type strain (Figure 3a). Conversely, in the *Hxt-Null* mutant, Snf1/AMPK phosphorylation remained high after the addition of glucose, without any significant decrease until 60 min (Figure 3a). These data demonstrate that if glucose import is prevented, Snf1/AMPK dephosphorylation is completely impaired.

The fact that glucose does not affect Snf1/AMPK phosphorylation in the *Hxt-Null* strain (Figure 3a) implies that the perception of extracellular glucose is not involved in the regulation of Snf1/AMPK activity. This is also consistent with the unaffected dephosphorylation of Snf1/AMPK in the *pde1∆cyr1∆yak1∆* strain (Figure 1c), where the sensing of extracellular glucose no longer impinges on the Ras/PKA pathway [7]. 

Once active, Snf1/AMPK is known to alter the transcription and the activity of metabolic genes and enzymes counteracting glucose catabolite repression [23]. In agreement with a previous report, and despite the high level of phosphorylation of Snf1/AMPK, the *Hxt-Null* strain was unable to grow when glucose and ethanol were mixed as carbon sources [21] (Figure 3b). Furthermore, our attempt to isolate *Hxt-Null* mutants able to consume ethanol in the presence of glucose failed. These results indicate that glucose catabolite repression predominates over the consumption of alternative carbon sources to glucose, even when the Snf1/AMPK regulatory mechanism is impaired.

### 2.3. G6P, and Not F1,6BP, Influences Snf1/AMPK Phosphorylation

The dependency of Snf1/AMPK phosphorylation on glucose transport rate (Figure 2) is consistent with the existence of a flux-sensing mechanism that coordinates cell signaling with glycolytic rate. F1,6BP concentration has been proposed to be the core of this mechanism, being proportional to the glycolytic flux [11]. In addition, in mammalian cells, F1,6BP controls the stability of the AXIN/LKB1/AMPK complex, thus regulating AMPK-T172 phosphorylation [14].

**Figure 4:**
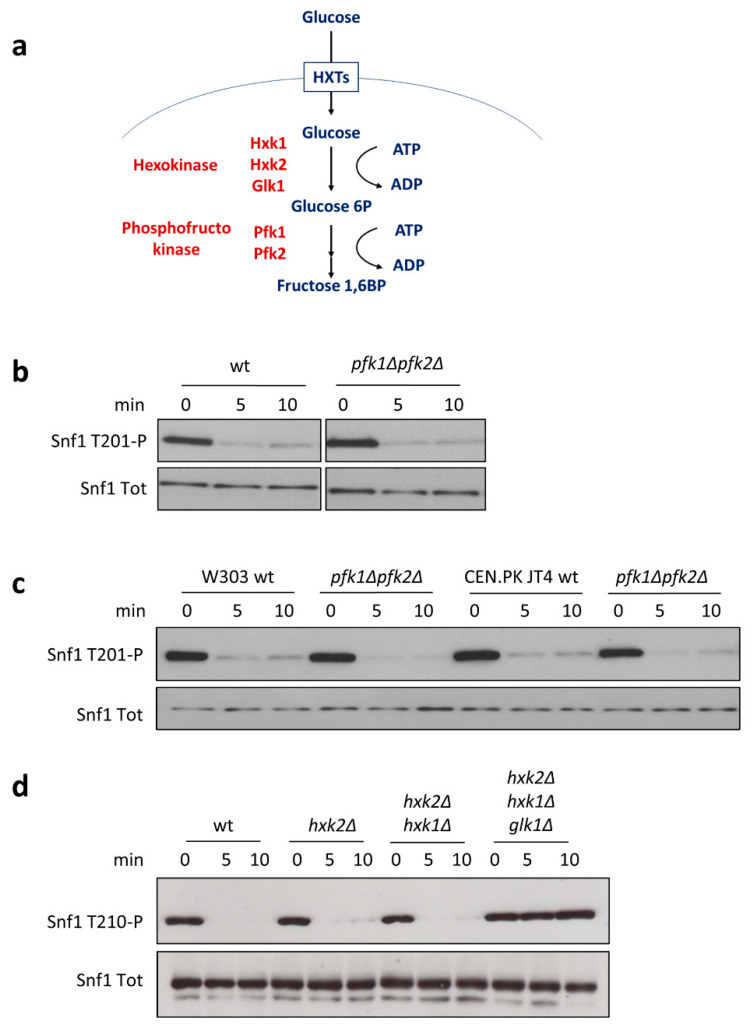
Snf1-T210 phosphorylation depends on G6P. (**a**) Schematic representation of the upper part of glycolysis in yeast. (**b**) Shift-up experiments in the *pfk1∆pfk2∆* strain, with the two genes coding for phosphofructokinase deleted. (**c**) Shift-up experiments in *pfk1∆pfk2∆* mutants in W303 and CEN.PK JT4 backgrounds. (**d**) Shift-up experiments in strains with one or more genes coding for hexokinases deleted. Anti-pAMPK antibody (to detect Snf1-T210 phosphorylation) and anti-His antibody (to detect total Snf1) were used. One representative immunoblot is shown.

As shown above, we excluded any effect of F1,6BP on the Snf1/AMPK phosphorylation state mediated by the Ras/PKA pathway (Figure 1). Because of this, we wondered whether F1,6BP may affect the Snf1/AMPK activation state through other mechanisms. To address this issue, we carried out shift-up experiments in a yeast strain in which both genes encoding for phosphofructokinase (*PFK1* and *PFK2*) were deleted to prevent the synthesis of F1,6BP from glucose [24] (Figure 4a). Surprisingly, the *pfk1∆pfk2∆* strain presented rapid and almost complete dephosphorylation of Snf1/AMPK upon the addition of glucose, as did the control strain (Figure 4b).

Yeast strains of the CEN.PK background have been proven to carry a point mutation in the *CYR1* gene, known to influence yeast metabolism upon the deletion of glycolytic genes [25]. To completely exclude side effects due to this mutation, we also conducted shift-up experiments in the W303 and CEN.PK JT4 backgrounds, bearing the wild-type *CYR1* gene [25]. Remarkably, in both the backgrounds, we observed complete dephosphorylation of Snf1/AMPK without phosphofructokinase activity and upon the addition of glucose (Figure 4c), indicating that F1,6BP is not involved in the control of the activation state of Snf1/AMPK. Thus, despite the inverse correlation between Snf1/AMPK phosphorylation and glucose uptake rate (Figure 2c), we failed to show a correlation between F1,6BP synthesis and Snf1/AMPK activation (Figure 4b,c). 

Next, we investigated the role of hexokinases (Hxk1, Hxk2, and Glk1), given that glucose-6-phosphate (G6P) synthesis was already suggested to influence Snf1/AMPK phosphorylation state [5]. Among yeast hexokinases, Hxk2 represents the catalytically dominant form, which also has a regulatory function involved in the maintenance of glucose repression [26]. Nevertheless, Snf1/AMPK dephosphorylation in the *hkx2∆* strain still responded to the addition of glucose, suggesting that Hxk2’s regulatory function is not involved in the control of Snf1/AMPK activation (Figure 4d). Similarly to *hxk2Δ* cells, the double-deleted *hxk1Δhxk2Δ* strain also showed rapid dephosphorylation of Snf1/AMPK upon the addition of glucose (Figure 4d), while only the triple-mutant *hxk1Δhxk2Δglk1Δ*, completely lacking hexokinase activity, resulted in a loss of Snf1/AMPK dephosphorylation (Figure 4d). These results are consistent with a recent publication showing that G6P—and not F1,6BP—concentration peaks within a few minutes after the addition of glucose [13]. 

Altogether, these data strongly indicate that G6P is the glycolytic intermediate that connects Snf1/AMPK phosphorylation with glucose metabolism.

## 3. Discussion

AMPK is a master regulator of glucose metabolism, as well as lipid metabolism, and in higher eukaryotes it plays crucial roles in several diseases, such as cancer, obesity, and diabetes [27]. Hypothalamic AMPK plays a critical role in whole-body energy homeostasis, mediating the effects of hormones and nutrients on food intake, energy expenditure, and metabolism of peripheral organs [28,29,30,31]. Indeed, AMPK activity is regulated by several compounds other than glucose, such as α-lipoic acid, leptin, insulin, ghrelin, cytokines, as well as natural compounds such as resveratrol, curcumin, epigallocatechin 3-gallate, and many others [32,33,34]. Among these, α-lipoic acid—a cofactor of mitochondrial enzymes with antioxidant capacity—was shown to decrease AMPK activity in the hypothalamus, leading to anti-obesity effects in different organisms [35,36]. On the other hand, α-lipoic acid was shown to increase AMPK activation in β-cells [37] and in the liver in eukaryotic systems [36].

Although AMPK modulation is much more complex in mammals, the use of model organisms to elucidate fundamental aspects of its regulation is a valuable tool to understand its activity in vivo. In particular, *Saccharomyces cerevisiae* biochemistry and genetics are extremely useful to study the crosstalk between different signaling pathways, as exemplified by this work.

**Figure 5:**
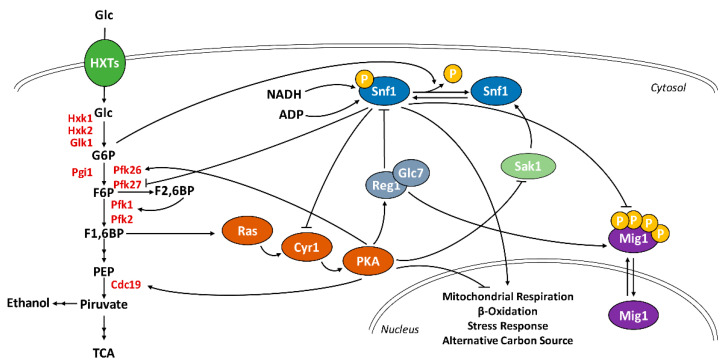
Schematic representation of the interactions between the glycolysis, Ras/PKA, and Snf1/AMPK pathways. F1,6BP synthesis influences Ras/PKA activity which, in turn, phosphorylates Pfk26 and Cdc19, stimulating the glycolytic flux. G6P promotes Snf1/AMPK dephosphorylation, thus inhibiting the kinase. NADH and ADP interact with Snf1/AMPK, giving rise to an interaction hub for the monitoring of cellular metabolism. Active PKA stimulates Reg1/Glc7 and reduces Sak1 activity, without influencing the Snf1/AMPK phosphorylation state, during a nutritional shift-up. In turn, phosphorylated Snf1/AMPK inhibits Cyr1 and Pfk27 activity and prevents Mig1 nuclear translocation. As a final result, PKA and Snf1/AMPK alternatively regulate the expression of genes involved in mitochondrial metabolism, stress response, and alternative carbon metabolism.

*S. cerevisiae* has evolved its metabolism to optimally consume glucose across a wide range of concentrations. This is achieved through a complex signaling network specialized for the sensing of glucose availability and for the dynamic adaptation of cellular metabolism [5]. The Ras/PKA pathway is strongly activated by glucose availability, and its function enhances cellular proliferation as well as glucose fermentation. PKA is also known to enhance the glycolytic flux, stimulating the pyruvate kinase Cdc19 and the 6-phosphofructo2-kinase Pfk26 through phosphorylation, triggering a positive feedback loop in which glucose availability stimulates glycolysis and simultaneously inhibits gluconeogenesis [38] (Figure 5).

Meanwhile, a glucose concentration higher than 0.05% represses Snf1-T210 phosphorylation, preventing the expression of genes involved in mitochondrial respiration, oxidation of fatty acids, and the consumption of alternative carbon sources to glucose [6]. Of note, PKA is also known to positively regulate the activity of the phosphatase Reg1/Glc7, responsible for Snf1/AMPK inactivation [16] (Figure 5). 

In the present work, we investigate the connection between Snf1/AMPK and Ras/PKA pathways during the onset of glucose catabolite repression (i.e., during a nutritional shift from ethanol to glucose). Our results clearly show that glucose induction of Snf1/AMPK dephosphorylation is completely independent of Ras/PKA activity, despite the dominant role of Ras/PKA in regulating yeast glucose metabolism (Figure 1). 

Signaling pathways are known to be organized with a bow-tie architecture, in which several signals are independently sensed by different signaling molecules, and then converge on a limited number of downstream effectors [39]. Thus, the Ras/PKA and Snf1/AMPK pathways are independently and alternatively influenced by glycolysis during a nutritional shift-up, while their signals can integrate on downstream effectors (Figure 5). An example of this concept is shown in Figure 3: even if Snf1/AMPK is highly phosphorylated in the *Hxt-Null* strain, cells robustly persist in the preference of glucose as carbon source, suggesting that glucose fermentation still predominates over Snf1/AMPK stimulation of ethanol consumption.

Glucose control of Snf1-T210 phosphorylation has long been considered to be dependent on glucose concentration in the growth medium [19]. To maximize glucose consumption, yeast cells have also evolved a signaling pathway controlling the expression of glucose transporters, in accordance with the availability of this nutrient. This regulation results in the matching of high glucose concentrations with the expression of the high-capacity transporters, whereas high-affinity transporters are expressed when the glucose concentration is low. Thus, glucose uptake rate is inherently dependent on glucose availability [4]. However, working with yeast strains constitutively expressing only one glucose transporter (either Hxt1, Hxt7, or Tm6*), we managed to decouple glucose transport rate from glucose availability in the medium. This allowed us to depict an inverse correlation between Snf1-T210 phosphorylation state and glucose transport rate, clearly showing that the activity of this kinase is dynamically regulated by glucose transport flux (Figure 2). Our results are also in agreement with previously reported microfluidic experiments, which proved an altered nuclear localization of Mig1 in the Hxt1-only and Hxt7-only strains [40]. Mig1 is a transcriptional repressor known to inhibit the expression of genes involved in alternative carbon metabolism. In turn, active Snf1/AMPK massively phosphorylates Mig1, promoting its Msn5-mediated export from the nucleus [41]. Moreover, the altered Mig1 nuclear localization in strains with a modified glucose transport indicates that glucose uptake rate influences the entire Snf1/AMPK pathway, alongside its downstream key metabolic effectors. Of note, a connection has recently been reported between mammalian AMPK and glycolytic metabolism [14], supporting consistent evolutionary conservation of that regulation. 

As recently reviewed, protein–metabolite interactions (PMIs) are molecular events pivotal for the coordination between cellular metabolism and signal transduction [12]. 

F1,6BP is one of the most influential metabolites, controlling both Ras/PKA activation in yeast and AMPK phosphorylation inhibition in mammalian cells, through a mechanism mediated by aldolases [13,14]. Moreover, sugar phosphates such as G6P, G1P, and T6P inhibit SnRK1 (the plant ortholog of AMPK) [42,43], suggesting evolutionary conservation of the role of sugar phosphates in the regulation of the AMPK kinases. In keeping with that, our data show that G6P regulates Snf1/AMPK phosphorylation; indeed, mutant strains completely lacking glucose transporters or hexokinases are unresponsive to the addition of glucose (Figure 3a and Figure 4d). However, we cannot rule out the possibility that this interaction is direct or mediated, since we did not succeed in measuring the interaction between G6P and the Snf1/AMPK complex through isothermal calorimetry analysis. 

We further explored the possibility of a direct interaction by considering the carbohydrate-binding properties of Snf1/AMPK β-subunits. Thus, we performed a docking analysis with several sugar phosphates on the glycogen-binding domain (GBD) of Sip2 (from PDB ID: 2qlv) or Gal83 (modelled by homology, based on Sip2-GBD). In fact, Gal83 GBD was previously reported to be required for the glucose-dependent dephosphorylation of Snf1 [44]. Unfortunately, this approach failed to detect a binding pocket with a high affinity for G6P. Thus, we considered the hypothesis of G6P acting on GBD when bound on the active complex. The conformation of the active complex in yeast is not available, but several structures of the active mammalian AMPK were resolved where the carbon-binding motif (CBM) of the β-subunit was docked on the active kinase domain, generating a pocket that can bind to several activators [45,46]. We attempted the construction of a homology model for the yeast GBD in the same conformation, but it was evident that whereas the kinase pocket facing CBM is conserved from mammalian to yeast, neither the GBD from Sip2 nor from Gal83 shares the characteristics of the mammalian homolog interface (see Supplementary Figure S1). This raises doubts as to whether this conformation could actually exist in yeast.

The only other structure available in yeast was obtained with the truncated C-terminal portion of Snf1, and shows the Sip2-GBD docked on a completely different site, bound to Snf4 [47]. This is not compatible with the presence of the kinase domain in the active complex, as characterized for mammalian AMPK complexes. In this yeast structure, we could identify potential binding pockets for G6P, although it is still unclear whether this conformation represents an alternative physiological state for the Snf1 complex, possibly reflecting the inactive conformation of Snf1. Thus, at the moment, only our in vivo data can strongly support the dependence of the Snf1/AMPK phosphorylation state on G6P. 

Therefore, metabolites of the upper part of glycolysis result in actively influencing the signaling pathways that regulate glucose metabolism in yeast, mammals, and plants [13,14,42,43]. The present work highlights that yeast Snf1/AMPK undergoes a similar regulation, with G6P being the molecule connecting the Snf1/AMPK phosphorylation state with glycolytic flux; indeed, this enables the monitoring of glucose metabolism and the coordination of fermentative and respiratory metabolism. In addition, through the interaction with ADP [48] (Figure 5), Snf1/AMPK senses the energetic status of the cell and regulates catabolic and anabolic processes to achieve energy homeostasis.

In conclusion, our observations, alongside previously reported interactions, underline the role of Snf1/AMPK as a central hub for the tuning of cellular metabolism through protein–metabolite interactions. 

## 4. Materials and Methods

### 4.1. The Yeast Strains and Growth Conditions

The *S. cerevisiae* strains employed in this work are listed in Table S1. Cells were grown at 30 °C in synthetic media containing 2% and 5% glucose (Merck Life Science, Milan, Italy) or 2% ethanol (Merck Life Science, Milan, Italy), 6.7 g/L yeast nitrogen base (Formedium, Hunstanton, England), and 50 mg/L of the required amino acids (Merck Life Science, Milan, Italy). 

For shift-up experiments, cells were grown overnight in 2% ethanol. At the mid-exponential phase (cellular concentration 0.5–0.8 OD/mL), glucose was added at a final concentration of 2%, and samples were collected at different timepoints, quenched, and extracted as reported below. The *ras2∆*, *tpk1∆tpk2∆tpk3∆yak1∆*, and *pde2∆cyr1∆yak1∆* strains, which are unable to grow on ethanol, were pre-cultured overnight in 2% glucose media and then transferred to 2% ethanol media for 2 h before the shift-up experiment. 

### 4.2. Protein Extraction, Immunoblotting, and Densitometric Analysis

To assess the Snf1/AMPK phosphorylation state, cells were quenched using 6% TCA (Merck Life Science, Milan, Italy). Then, the cells were lysed in UREA buffer (6 M UREA, 1% SDS, 50 mM Tris-HCl pH 7.5, 5 mM EDTA) in the presence of an equal volume of acid-washed glass beads (Merck Life Science, Milan, Italy) by 10 vortex/ice cycles of 30 sec each. Crude extracts were denatured in SDS sample buffer (40% glycerol, 20% β-mercaptoethanol, 9.2% SDS, 0.25 mM Tris-HCl pH 6.8, 0.01% BBF) and heated for 5 min at 95 °C. 

Western blot analysis was conducted with anti-phosphoT172-AMPK antibody (Cell Signaling, Danvers, MA, USA), anti-His antibody (Merck Life Science, Milan, Italy), and anti-TAP antibody (Thermo Fisher Scientific, Waltham, MA, USA). Densitometric analyses were conducted with ImageJ software v1.51 (NIH, http://imagej.nih.gov/ij/, accession date 23 April 2018).

### 4.3. Glucose Consumption Rate

Cells in the exponential phase of growth were sampled by centrifugation, collecting the supernatants. Glucose concentration at each timepoint was measured using the D-Glucose HK/G6P DH Kit (Megazyme, Bray, Ireland), according to the manufacturer’s instructions. 

Glucose consumption rate (mmol/g × h) was calculated as the angular coefficient of the correlation between glucose concentration (mmol/mL) and cellular concentration, corrected for the cellular dry weight (mg/mL) and time (h). 

### 4.4. Statistical Analysis

Experiments were performed in triplicate. Results were compared by using Student’s two-sided *t*-test. Differences were considered statistically significant at *p* < 0.05.

## Data Availability

The data presented in this study are available on request from the corresponding authors.

## Supplementary Materials

The following are available online at https://www.mdpi.com/article/10.3390/ijms22179483/s1.
