# Supplementary material for: AMPK Phosphorylation Is Controlled by Glucose Transport Rate in a PKA-Independent Manner
Source: Int J Mol Sci. 2021 Aug 31;22(17):9483. doi: 10.3390/ijms22179483 (PMC8431435; doi:10.3390/ijms22179483)
Supplement: Supplementary file 1 [file ijms-22-09483-s001.zip › ijms-1336701-supplementary.pdf]

Supplementary information

AMPK phosphorylation is controlled by glucose transport rate in a PKA-independent manner

Riccardo Milanesi, Farida Tripodi, Jacopo Vertemara, Renata Tisi, Paola Coccetti

Supplementary Table S1

Strain	Genotype	Source
W303 1a		OpenBiosystem
CDC25^{T1490P}	W303-1a *cdc25::CDC25^{T1490P}*	Peeters et al., 2017
ras2Δ	BY472 *ras2::KanMX*	This work
tpk1Δtpk2Δtpk3Δyak1Δ	*W303-1a tpk1::ADE8 tpk2::HIS3 tpk3::TRP1 yak1::LEU2 ura3-52 leu2-3,112 trp1 ade8*	Van De Velde et al., 2008
pde1Δcyr1Δyak1Δ	*W303-1a pde2::TRP1 cyr1::KanMX yak1::LEU2*	Van De Velde et al., 2008
Hxt-Null	CENPK2C MATa *MAL2-8c SUC2 hxt17 ura3-52 gal2::loxP stl1::loxP agt1::loxP ydl247w::loxP yjr160c::loxP hxt13::loxP hxt15::loxP hxt16::loxP hxt14::loxP hxt12::loxP hxt9::loxP hxt11::loxP hxt10::loxP hxt8::loxP hxt514::loxP hxt2::loxP hxt367::loxP*	Elbing et al., 2004
Hxt1 only	HXT7prom-HXT1-HXT7term *ura3-52::URA3*	Elbing et al., 2004
Hxt7 only	HXT7prom-HXT7-HXT7term *ura3-52::URA3*	Elbing et al., 2004
Tm6*	HXT7prom-TM6(HXT1 bp1-741, HXT7 bp742-1713)-HXT7term *ura3-52::URA3*	Elbing et al., 2004
CENPK2C	*leu2-3 ura3-52 trp1-2898 his3-1 MAL2-8C SUC2 HXT17*	Elbing et al., 2004
pfk1Δpfk2Δ	CENPK2C *pfk1::KanMX pfk2::HPH*	This work
CENPK JT4	Mat*a LCR1*	Kummel et al., 2010
pfk1Δpfk2Δ	CENPK JT4 *pfk1::KanMX pfk2::HPH*	This work
pfk1Δpfk2Δ	W303-1a *pfk1::KanMX pfk2::HPH*	This work
Snf1-TAP	BY4741 *SNF1-TAP his3Δ1 leu2Δ0 met15Δ0 ura3Δ0*	OpenBiosystems

a

```
AAKB2_HUMAN      ARPTVIRWSEGGKEVFISGSFNNWSTKIPLIK---SHNDFVAILDLPEGEHQYKFFVDGQ  132
AAKB1_RAT        ARPTVFRWTGGGKEVYLSGSFNNWS-KLPLTR---SQNNFVAILDLPEGEHQYKFFVDGQ  132
SIP2_YEAST       MVPVEIRWQQGGSKVYVTGSFTKWRKMIGLIPDSDNNGSFHVKLRLLPGTHRFRFIVDNE  222
GAL83_YEAST      MFPVDITWQQGGNKVYVTGSFTGWRKMIGLVPVPGQPGLMHVKLQLPPGTHRFRFIVDNE  220
                   *. : *  **.:*:::***. *   : *     . . : . * *  * *:::*:**.:

AAKB2_HUMAN      WVHDPSEPVVTSQLGTINNLIHVKKSDFEVFDAL---------KLDSMESSE--------  175
AAKB1_RAT        WTHDPSEPIVTSQLGTVNNIIQVKKTDFEVFDAL---------MVDSQKCSD--------  175
SIP2_YEAST       LRVSDFLPTATDQMGNFVNYIEVRQPEKNPTNEK--IR------SKEADSMRPPTSDRSS  274
GAL83_YEAST      LRFSDYLPTATDQMGNFVNYMEVSAPPDWGNEPQQHLAEKKANHVDDSKLSKRPMSARSR  280
                    .   *  .*.*:*.. * :.*        :             .. .
```

b

```
SNF1_YEAST       MSSNNNTNTAPANANSSHHHHHHHHHHHHHGHGGSNSTLNNPKSSLADGAHIGNYQIVKT  60
AAPK1_HUMAN      ----------------------------MRRLSSWRKMATAEKQKHDGRVKIGHYILGDT  32
AAPK1_RAT        ----------------------------MRRLSSWRKMATAEKQKHDGRVKIGHYILGDT  32
                                              :  .. ..  .  *..  . .:**:* : .*

SNF1_YEAST       LGEGSFGKVKLAYHTTTGQKVALKIINKKVLAKSDMQGRIEREISYLRLLRHPHIIKLYD  120
AAPK1_HUMAN      LGVGTFGKVKVGKHELTGHKVAVKILNRQKIRSLDVVGKIRREIQNLKLFRHPHIIKLYQ  92
AAPK1_RAT        LGVGTFGKVKVGKHELTGHKVAVKILNRQKIRSLDVVGKIRREIQNLKLFRHPHIIKLYQ  92
                 ** *:*****:. *  **:***:**:*:: : . *: *:*.***. *:*:*********:

SNF1_YEAST       VIKSKDEIIMVIEYA-GNELFDYIVQRDKMSEQEARRFFQQIISAVEYCHRHKIVHRDLK  179
AAPK1_HUMAN      VISTPSDIFMVMEYVSGGELFDYICKNGRLDEKESRRLFQQILSGVDYCHRHMVVHRDLK  152
AAPK1_RAT        VISTPSDIFMVMEYVSGGELFDYICKNGRLDEKESRRLFQQILSGVDYCHRHMVVHRDLK  152
                 **.: .:*:**:**. *.****** :..::.*:*:**:****:*.*:***** :******
```

Supplementary Figure S1. Yeast β-subunits do not share the interface to AMPK kinase domain identified in mammalian homologs. Sequences of the indicated proteins were aligned with Clustal Omega. The blue boxes highlight the residues involved in the interface between the CBM of the β-subunit (panel a) and the AMPK kinase domain (panel b) in rat AMPK structure (PDB ID:4qfg).

Supplementary Materials and methods (for Supplementary Figure S1)

The whole sequences from AAKB2_HUMAN, AAKB1_RAT, SIP2_YEAST, GAL83_YEAST, and SNF1_YEAST, AAPK1_HUMAN and AAPK1_RAT entries from UniprotKB database were submitted to Clustal Omega (https://www.ebi.ac.uk/Tools/msa/clustalo/) in order to obtain a reliable multiple sequence alignment. Only the partial alignment containing residues involved at the interface between the CBM/GBD of the β–subunit and the AMPK kinase domain were shown in the Supplementary Figure S1. These residues were identified by an analysis on rat AMPK complex structure (PDB ID: 4qfg) since it is the only structure available with no activators positioned at the interface. The analysis of the contact residues was performed by UCSF Chimera software, by imposing a threshold of 4 Å from the interacting partner.
